# RhoA- and Actin-Dependent Functions of Macrophages from the Rodent Cardiac Transplantation Model Perspective -Timing Is the Essence

**DOI:** 10.3390/biology10020070

**Published:** 2021-01-20

**Authors:** Malgorzata Kloc, Ahmed Uosef, Martha Villagran, Robert Zdanowski, Jacek Z. Kubiak, Jarek Wosik, Rafik M. Ghobrial

**Affiliations:** 1 The Houston Methodist Research Institute, Houston, TX 77030, USA; auosef@houstonmethodist.org (A.U.); RMGhobrial@houstonmethodist.com (R.M.G.); 2 Department of Surgery, The Houston Methodist Hospital, Houston, TX 77030, USA; 3 M.D. Anderson Cancer Center, Department of Genetics, The University of Texas, Houston, TX 77030, USA; 4 Electrical and Computer Engineering Department, University of Houston, Houston, TX 77204, USA; mvillagran@houstonmethodist.org (M.V.); jarek@uh.edu (J.W.); 5 Texas Center for Superconductivity, University of Houston, Houston, TX 77204, USA; 6 Laboratory of Molecular Oncology and Innovative Therapies, Military Institute of Medicine (WIM), 04-141 Warsaw, Poland; rzdanowski@wim.mil.pl; 7 Department of Regenerative Medicine and Cell Biology, Military Institute of Hygiene and Epidemiology (WIHE), 01-163 Warsaw, Poland; jacek.kubiak@univ-rennes1.fr; 8 Cell Cycle Group, CNRS, Faculty of Medicine, Institute of Genetics and Development of Rennes, University of Rennes, UMR, 6290 Rennes, France

**Keywords:** RhoA, ROCK, Rac1, actin, macrophage, transplantation, mouse, rat, timing, circadian rhythm, chronic rejection

## Abstract

**Simple Summary:**

The functions of animal and human cells depend on the actin cytoskeleton and its regulating protein called the RhoA. The actin cytoskeleton and RhoA also regulate the response of the immune cells such as macrophages to the microbial invasion and/or the presence of a non-self, such as a transplanted organ. The immune response against transplant occurs in several steps. The early step occurring within days post-transplantation is called the acute rejection and the late step, occurring months to years post-transplantation, is called the chronic rejection. In clinical transplantation, acute rejection is easily manageable by the anti-rejection drugs. However, there is no cure for chronic rejection, which is caused by the macrophages entering the transplant and promoting blockage of its blood vessels and destruction of tissue. We discuss here how the inhibition of the RhoA and actin cytoskeleton polymerization in the macrophages, either by genetic interference or pharmacologically, prevents macrophage entry into the transplanted organ and prevents chronic rejection, and also how it affects the anti-microbial function of the macrophages. We also focus on the importance of timing of the macrophage functions in chronic rejection and how the circadian rhythm may affect the anti-chronic rejection and anti-microbial therapies.

**Abstract:**

The small GTPase RhoA, and its down-stream effector ROCK kinase, and the interacting Rac1 and mTORC2 pathways, are the principal regulators of the actin cytoskeleton and actin-related functions in all eukaryotic cells, including the immune cells. As such, they also regulate the phenotypes and functions of macrophages in the immune response and beyond. Here, we review the results of our and other’s studies on the role of the actin and RhoA pathway in shaping the macrophage functions in general and macrophage immune response during the development of chronic (long term) rejection of allografts in the rodent cardiac transplantation model. We focus on the importance of timing of the macrophage functions in chronic rejection and how the circadian rhythm may affect the anti-chronic rejection therapies.

## 1. Introduction

Over the years, organ transplantation became the ultimate savior for patients with a fatal organ failure. Although the extraordinary progress in the surgical techniques, development of new organ preservation methods, and immunosuppression therapies improved the short-term functioning and survival of the transplanted organs, the long-term organ fitness and survival remain an unresolved hurdle. Approximately 10 years post-transplantation, between 50 to 70% (depending on the type of the organ) of transplants fail because of the chronic (long-term) rejection [1]. For example, the official statistics show that 97% of kidney transplants are fully functioning after one month, 93% after 1 year, and 83% at 3 years post-transplantation. Some transplantation centers achieved a 55.6% survival rate of heart transplants after 20 years. The patients who rejected the organ require retransplantation. They come back to the transplantation list and exacerbate a shortage of donated organs. The resolution of these problems requires further studies in the transplantation model systems. Because of the prohibitive cost of large animals, the most popular models to study the cellular and molecular aspects of organ rejection are rodents (mouse and rats) transplantation models [2] Additionally, the rodents, and especially mice can be easily manipulated genetically, which allows one to study the function of the particular gene(s) and/or the immune cell subsets and define their exact function in transplant rejection. The rodent transplantation models are also invaluable tools in a wide screening of anti-rejection drugs.

Although the adaptation and translation of the results and conclusions from the rodent studies to humans are limited by the interspecific differences, and the great disparity in the lifespan, rodent models can provide the basis for eventual clinical testing in humans. Another limitation is that the therapeutic drugs used in the animal models are usually unapproved for human use. Thus, translation of the animal results to the clinic presents a challenge of finding the clinically approved drugs, with the desired functions, for the animal testing. A very good example is our search for clinically applicable anti-chronic rection drugs. The results of our studies in the rodent transplantation model showed that many commercially available—but clinically unapproved—RhoA/ROCK inhibitors were very effective in the prevention of chronic rejection of the transplants. Searching for the clinically approved RhoA inhibitors we found that the drugs commonly used for the treatment of multiple sclerosis (MS), Fingolimod, and Siponimod also inhibit the RhoA. Fingolimod and Siponimod are the modulators of sphingosine 1-phosphate receptors (S1P). The sphingosine, a 2-amino-1,3-dihydroxy-octadec-4-ene, is a component of the group of cellular lipids called the sphingolipids. Out of all mammalian tissues, the nervous system contains the highest concentration of sphingolipids. Because of this, the S1P receptors became one of the favored therapeutic targets for the treatment of the nervous system diseases. Although Fingolimod and Siponimod are chemically similar, the Fingolimod is only beneficial for the relapsing MS, while the Siponimod reduces progression of disability in secondary progressive MS (SPMS). Thus, after proving the efficacy of Fingolimod in the prevention of chronic rejection in the animal models, this drug can be readily transposed to clinical trials.

Chronic rejection is a very complicated, cellularly and molecularly multifactorial, and is a process still not fully understood, in which the macrophages are major players involved in the development of graft tissue fibrosis and occlusion of its blood vessels. It is known that macrophages induce the smooth muscle cells, which are the component of the blood vessel wall, to overproliferate. This, in turn, causes a reduction of the vessel lumen, and eventually, a complete closure of the lumen. Additionally, some of the macrophage-released signaling factors induce graft fibroblasts to over-produce collagen fibers, which destroys graft tissue integrity [3,4]. Besides the opportunity to study and manipulate macrophage immune response to transplantation, the rodent transplantation models are also very useful for the studies of universal macrophage functions and their regulatory molecules, far beyond the bounds of transplantation.

## 2. Actin-Dependent Functions of the Macrophages

Depending on the requirements and signaling from the cellular and acellular microenvironment they receive, macrophages can adjust their metabolic and functional phenotype, and play homeostatic, anti-inflammatory, or pro-inflammatory roles [5,6]. The majority of macrophage functions important for the immune response in transplantation, and beyond, are actin-dependent. It is well established that actin polymerization and polymerization/depolymerization dynamics are regulated by the small GTPase RhoA and Rac1, their downstream effectors, and the interacting pathways, such as guanine nucleotide exchange factors (GEFs), and a rapamycin-insensitive protein complex 2 (mTORC2) [7,8]. Because different Rho GTPases affect each other reciprocally and with interacting pathways, the specific regulatory molecules involved in actin dynamics may differ between cell types, studied processes, and experimental settings. Below we describe actin-dependent processes crucial to macrophage function.

## 3. Phagocytosis

**Figure 1:**
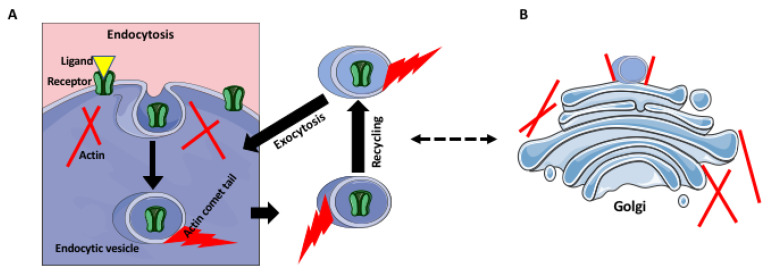
Actin in receptor recycling and Golgi. (**A**) An “used” ligand-bound receptor is internalized by endocytosis. The ligand is released in the acidic interior of the late endosome, and sorted into the lysosome-fusing vesicles. The receptors are sorted into the recycling vesicles and return to the cell membrane. The endocytic vesicles may move using actin comets, which are blocked by the GTPase inhibitors. (**B**) Some surface receptors are also recycled through the Golgi complex, which can exchange components with the endosomal pathway. The Golgi cisternae and budding vesicles are supported and anchored by the actin filaments.

Phagocytosis, i.e., an engulfment of microbes, dying cells, and cell/tissue debris, is essential for the immune defense and tissue remodeling/homeostatic functions of macrophages. Phagocytosis requires an extensive remodeling of the cell membrane, which allows the formation of the phagocytic cup and phagosomes. Such membrane remodeling depends on the actin and its RhoA and/or Rac1 dependent dynamics [9,10,11]. Mammalian macrophages initiate phagocytosis by recognizing the foreign object coated (opsonized) by the immunoglobulins (Igs) or the complement. The recognition of the conserved Fc domain of Igs by the macrophage Fc receptors induces actin-dependent extension of the cell membrane resulting in the enclosure/internalization of the target [9]. This process involves the recruitment and activation of tyrosine kinase (spleen tyrosine kinase) SYK, which transmits signals leading to actin polymerization and the closure of phagocytic cup; the SYK-knockout macrophages cannot internalize the target [9,12,13]. The phagocytosis of the complement-bound particles, which is mediated by the CR receptors, also requires actin polymerization, but does not involve extensive membrane remodeling [9]. Besides the Igs and complement, a broad spectrum of other molecules (for example, fibronectin, lipopolysaccharides (LPS), lectins, and pathogen-associated molecular patterns (PAMPs), which have relevant receptors on the macrophages, can initiate phagocytosis [14]. Similar to Fc receptor-mediated phagocytosis, these receptors also induce a profound actin-dependent remodeling of the membrane [9]. Actin not only participates in the target internalization and formation of the phagosome, but also the movement of the phagosomes within the cytoplasm. The video microscopy studies of the phagosomes within the cytoplasm of the bone marrow macrophages showed that their movement is driven by the actin-rich rocket tails [15]. Such actin-propelled movement, driven by the assembly of the actin comet-tails has been also described for the membranous vesicles in vitro, and endosomes in vivo (Figure 1). In [9,16,17]. Thus, because many steps of phagocytosis depend on actin, they will be affected by the RhoA pathway interference [14]. Interestingly, a subtype of phagocytosis, the so-called clearance phagocytosis that is triggered by dead or dying cells’ signals, such as an exposed inner leaflet lipid phosphatidylserine (PS), which are recognized by the death receptors (DRs), although also actin-dependent in the formation of the phagocytic cup, requires Rac1 activity, and is inhibited by the RhoA [14]. Studies on the bone marrow-derived macrophages and macrophage J774 cell line showed that the clearance phagocytosis is enhanced by inhibition of RhoA, or its downstream effector ROCK [18,19].

## 4. Receptor Trafficking and Recycling

Similar to phagocytosis, receptor trafficking and recycling are also actin-dependent. The receptor turnover within the cell consists of endocytosis, endosomal recycling, the exocytic delivery of the recycled and renewed receptors to the cell surface, and their final insertion into the cell membrane (Figure 1). All these steps involve actin cytoskeleton and upstream signaling pathways that regulate actin polymerization and depolymerization and generate forces necessary for membrane deformation and vesicle movement [20,21,22,23]. There are also studies showing that endosome vesicle movement occurs via the actin comet tails that require the function of GTPases (Figure 1). Because the receptor recycling depends on the endocytic pathway, it also depends on the proper organization and functioning of the Golgi apparatus, which regulates endosome trafficking (Figure 1), In [24,25,26]. The Golgi apparatus consists of the perinuclearly located stack of cisternae, which bud off the transporting vesicles. The structural integrity and the proper formation of the endocytic vesicles depend on the Golgi-associated actin filaments and their regulators, such as the RhoA pathway [27]. Thus, any disruption of Golgi positioning and structure will also affect endosomes and receptor trafficking.

## 5. Tunneling Nanotubes (TNTs)

**Figure 2:**
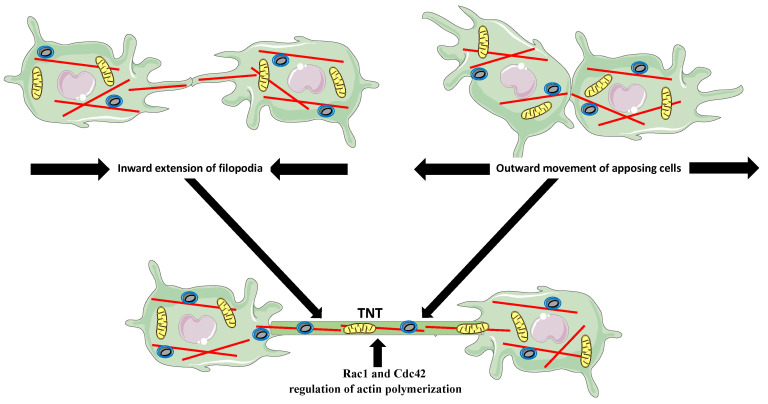
Two modes of tunneling nanotubes (TNTs) formation. The distant cells extend filopodia, which fuse and elongate to form the TNT (**upper left panel**). The closely apposing cells attach and move outward extending the membrane connection into a TNT (**upper right panel**). The bottom panel shows two cells connected by TNT, which transports organelles, such as mitochondria and vesicles, between cells.

Tunneling nanotubes are membranous, actin-based, and sometimes also contain microtubules, the channels between spatially distant cells that facilitate the long-distance exchange of vesicles, signaling molecules, and chiefly, the large organelles (Figure 2) [28,29,30,31,32]. For example, the observed both in vivo and in vitro transfer of mitochondria from the mesenchymal stem cells, improves macrophage energy production and phagocytosis [33]. Studies showed that the TNT-based exchange between macrophages and tumor cells promotes tumor progression and metastasis [30]. The super-resolution imaging and the time-lapse studies of immune cells, including human and RAW/L5 macrophages, suggest that the TNTs can form in two different ways. Either the distant cells form the actin-dependent protrusions, which meet and fuse, or the closely apposed cells fuse their membranes and move away, extending the fused region into a tube (Figure 2) [29,34,35,36,37]. Rho GTPases’ inhibition studies showed that the inhibition of Rac1 and Cdc42, which regulate Arp2/3-dependent actin polymerization, significantly decreased the frequency and longevity of TNTs formation in the bone marrow-derived and RAW macrophages [29].

## 6. Cell Morphology and Locomotion

The shape and morphology of eukaryotic cells depend on the function they perform and motility status, and vice versa, the function determines the morphology and motility. Studies using micropatterning to change macrophage shape showed that a mechanical elongation of the pro-inflammatory M1 macrophages leads to the expression of anti-inflammatory markers typical for the M2 phenotype [38]. The combination of computer simulations, mathematical analyses, and in vitro experiments showed recently that the orientation of actin stress fibers influences the geometry of the cell edge and cell shape [39,40]. Actin also participates in the positioning of the organelles within the cell, holding them non-random, strictly defined, and dependent on the functional and cell cycle status and needs. In turn, the changes in the position of the nucleus affect cell functions and signaling, and the genetically inherited defects in anchoring genes lead to many human diseases [41]. Actin’s role in the positioning of the cell nucleus is twofold; it forms a stable anchoring cytoskeleton or supplies active force for repositioning of the nucleus. Studies in *C. elegans* and mice showed that the nuclear envelope protein with an Unc84 (SUN) domain, the (UNC-84/SUN), recruits the actin-binding protein Syne/ANC-1, which attaches the nuclear envelope to the actin filaments. During the repositioning of the nucleus, the UNC-84/SUN interacts with lamin and transfers the propelling force from the cytoplasm cytoskeleton to the nucleoskeleton [42,43]. In the mouse, the knockout of the SUN1 protein disrupts nuclear anchoring [44]. Actin also plays a role in the shaping and anchoring of the mitochondria [45], and in cooperation with microtubules moves (to fulfill, for example, local energy demands) the mitochondria within the cells [46].

The forward movement of the macrophages occurs in several steps: the extension of the lamellipodia at the leading edge, strengthening the adhesion to the substrate at the front, translocation of the cell body, disassembly of adhesions and retraction of the trailing end, and finally recycling of the membrane and receptors from the rear to the front [47,48]. Cell morphology and motility depend on the actin-cytoskeleton regulated by Rho GTPases, including RhoA. The formation of a protruding edge is regulated by Rac, which induces the formation of F-actin, and the retraction of the trailing end by RhoA [8]. The real-time chemotaxis studies showed that peritoneal macrophages isolated from Rho A/B/C-deficient mice were unable to coordinate the detachment and retraction of the tail with the forward movement, which resulted in the extreme elongation of the tail. These macrophages also moved faster than control macrophages [49]. Authors suggested that in the in vivo situation, the pan-Rho-deficient macrophages could be recruited faster to the source of inflammation. Although this is certainly possible, authors have not taken into consideration the overall actin defects, which would disrupt other movement processes, such as the receptor expression and the ability to sense the target. We address this issue in more detail below in the transplantation-related paragraph.

## 7. Extracellular Matrix Degradation

**Figure 3:**
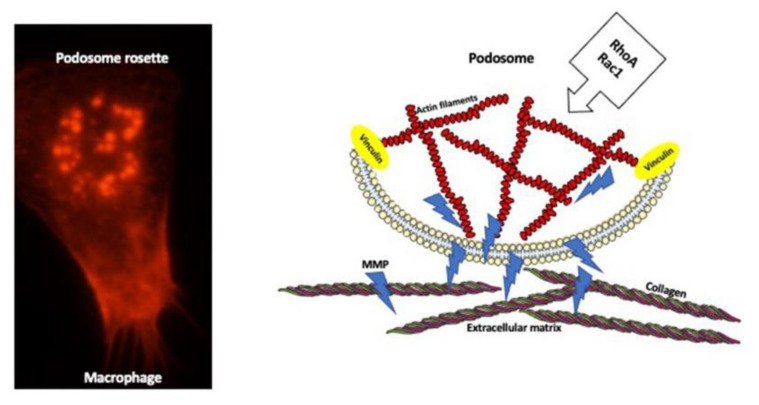
Podosome structure and function. Microscope image of mouse macrophage stained for actin (red color) shows podosomes arranged into a rosette (**left panel**). The diagram of the longitudinal section through the podosome (**right panel**) depicting the actin filament core surrounded by the adhesion molecules such as vinculin, which anchor actin filaments at the membrane. Podosome delivers matrix metalloproteinase enzymes (MMPs) that digest extracellular matrix components such as collagen.

Extracellular matrix (ECM) degradation and modeling, and basement membrane transmigration, are prerequisites for the fulfillment of macrophage functions in tissue regeneration, healing, and movement through the tissues [50]. The macrophage surface contacting the extracellular matrix has special dot-like organelles, called the podosomes, which deliver the matrix–lytic proteases (Figure 3).

Depending on the need, a single macrophage can have between 10 and 100 podosomes, which either are distributed randomly or organized in the super-structure, the podosome rosettes, which degrade the larger surface of ECM. Podosomes deliver various degradation enzymes, including several types of proteases: serine and cysteine proteases, metalloproteases, and the serine protease plasmin-activation (PA) system [51,52]. Podosomes contain an F-actin core surrounded by the ring of adhesion proteins [51,52,53]. They are highly dynamic structures, which assemble/disassemble within 2 to 12 min after formation. The podosome formation and dynamics are regulated by a variety of Rho GTPases including RhoA [52,53,54]. Recent studies showed that podosome actin has a very complex modular nano-architecture enabling podosome formation and mechanosensing of the microenvironment. The central core of the podosome is built of a central module of branched actin surrounded by a module of linear actin. Each module contains specific actin isoforms and interacting proteins. The central core is connected to two actin modules: ventral filaments linked by vinculin and attached to the cell membrane, and dorsal inter-podosomal filaments linked by myosin IIA. Depending on the substrate, the actin modules expand or shorten, mediating either the exploration and degradation of the substrate or short-range focal connectivity without degradation, respectively [55]. Not surprisingly, because of the roles of actin in podosome assembly and functions, the interference with actin regulator RhoA affects podosomes. Recent studies showed that podosome formation relies on the local inhibition of RhoA activity, and activation of Rac-1 [56,57]. The over-inhibition of RhoA causes podosome amplification [52], while the activation of RhoA and deactivation of Rac-1 cause podosome disassembly [57].

Although all the above-described actin-dependent macrophage functions rely on the actin filaments localized in the macrophage cytoplasm, we must not forget that a large pool of cellular actin is localized in the macrophage nucleus. As we will describe in the next section, the importance of nuclear actin for the regulation of crucial cellular (both nuclear and cytoplasmic) processes cannot be overestimated. Thus, we must remember that any interference with the RhoA pathway and actin assembly and dynamics will affect not only cytoplasmic but also nuclear actin and affect the related functions.

## 8. Nuclear Actin

**Figure 4:**
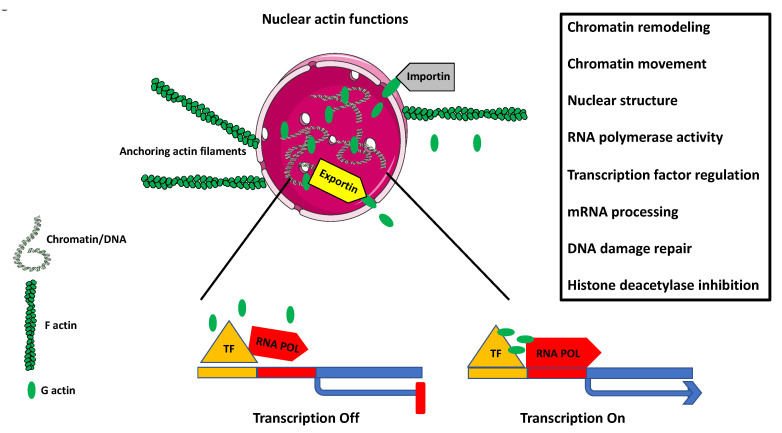
Nuclear actin. The diagram depicts some of the known functions of nuclear actin. The cell nucleus is anchored in q specific place within the cytoplasm by the cellular cytoskeleton containing actin filaments (F actin). The entry of the globular actin (G actin) to the nucleus is facilitated by the karyopherin protein Importin, and the exit is facilitated by the karyoprotein Exportin. Nuclear G actin plays a role in chromatin remodeling and chromatin movement. By regulating the activity of RNA polymerases I, II, III, and transcription factors, it regulates gene expression. It also participates in mRNA processing and DNA repair, and inhibits histone deacetylation. Additionally, nuclear matrix-associated actin plays a structural role in the overall nuclear organization.

After over 50 years of denial of existence of the nuclear actin and treating it as a procedural (immunostaining or extraction/isolation) artifact, science not only accepted the actin as a genuine component of the eukaryotic cell nucleus, but discovered its overreaching regulatory functions in the genomic, nuclear, and cytoplasmic processes [58,59,60,61]. It is now well established that nuclear actin is a component of chromatin remodeling complexes, and by binding RNA polymerase complexes and various transcription factors regulates gene expression, sequesters transcriptional activators and repressors, controls transcriptional/nuclear reprogramming, cell differentiation, and developmental reprogramming (Figure 4); In [61,62,63,64].

Although based on this information, it should be evident that nuclear actin must play a crucial role in the differentiation and activation of macrophages, the data on the subject are extremely limited. The chromatin immunoprecipitation on-chip assay, the genome-wide mapping of actin-binding to the gene promoters, and gene ontology analysis showed that during the phorbol 12-myristate 13-acetate (PMA)-induced differentiation of HL-60 cells into macrophage fate, actin regulates macrophage activation-related genes, including transactivation of the proton-coupled divalent metal ion transporters family gene, Slc11a1 [65]. This gene is necessary for macrophage activation, controls resistance to infection by sequestration of Fe(2+) and Mn(2+) cofactors of catalases and superoxide dismutases, protects macrophage against ROS, and limits the availability of cations used by microbes to synthesize the protective enzymes [66,67].

In the following section, we summarize how some of the above-described macrophage functions relate to the transplantation setting.

## 9. Transplantation Model and Methods to Study Macrophages

The research in our laboratory has been for many years focused on finding clinically applicable therapy for the chronic rejection of transplanted organs. We have used rat and mouse cardiac allograft transplantation models, in which the heart from the rodent host is transplanted heterotopically into the abdomen of the genetically disparate recipient [53]. The ascending aorta and the pulmonary artery of the donor’s heart are anastomosed to the recipient’s infrarenal aorta and inferior *vena cava*, respectively. In this model, the general health status (strength of the beating) of the transplanted heart is assessed daily by the palpation of the recipient’s abdomen [68]. The transplant is considered as non-rejected when it survives 100 days or longer, post-transplantation. However, transplant survival does not necessarily mean that the organ is healthy and has not undergone some degree of chronic rejection. Thus, the health status of the transplant and the degree, if any, of chronic rejection has to be assessed by following histopathology analyses. To study the role of specific genes and/or cell types, the recipient can be modified, before transplantation, using the constitutive (conventional or whole-body) knockout, the conditional (tissue-specific or inducible) knockout, or the protein function knockout when the gene of interest is substituted with a loss-of-protein function mutation [69]. In addition, the specific genes can be deleted, using the Cre/Lox recombination system from the specific cell types, such as macrophages [4,70]. Another approach, although time-consuming and technically challenging, which allows labeling, visualizing, and tracing of the desired cells in vivo, is a genetic manipulation leading to the expression of fluorescent proteins [71]. The macrophage localization within the graft and protein expression can be studied, at different time points post-transplantation, in the frozen or paraffin sections of the transplanted organ, using conventional histology staining and immunostaining with the antibodies against desired proteins (and/or macrophage markers). Additionally, at different time points, the monocytes/macrophages can be isolated from the blood, peritoneal cavity, or bone marrow and study for gene expression (at RNA and protein levels) using flow cytometry, RT-PCR, or immunostaining. Another approach to study the functions of macrophages and their response to different treatments (such as experimental anti-rejection drugs/inhibitors) is the isolation, usually from the non-transplanted, control recipient, of peritoneal or bone marrow monocytes/macrophages, and treatment in vitro in the cell culture. Subsequently, the in vitro grown and treated macrophages can be analyzed using the method(s) chosen from a vast array of available cellular and molecular techniques.

**Figure 5:**
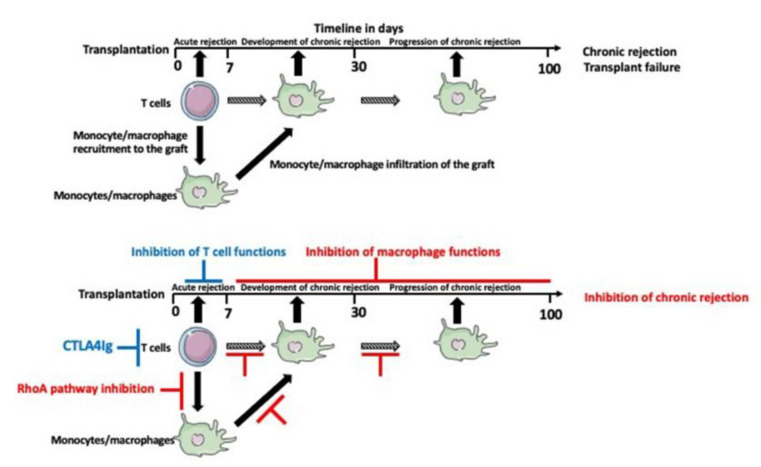
Timeline of transplant rejection and therapeutic intervention in the mouse cardiac transplantation model. The acute rejection, which occurs a few days after transplantation depends mainly on the T cells. The immunosuppressive drugs targeting T cells such as CTLA4Ig (in the mouse model) inhibit acute rejection but not the chronic rejection, which relies mainly on the macrophages. Administration of the RhoA pathway inhibitors within the first week of post-transplantation inhibits macrophage recruitment into the graft and inhibits chronic rejection.

Our interest in RhoA and actin-related macrophage functions in transplantation response was incited by our findings from RNA microarray analysis that chronically rejecting rat heart allografts upregulate RhoA [72]. Based on this finding, we hypothesized that the inhibition of the RhoA pathway in the transplant recipient might slow down or eliminate the development of chronic rejection of transplanted organs. Indeed, we showed that the commercially available RhoA pathway inhibitors, such as Y27632, Fasudil, Azaindole, or clinically approved for the treatment of multiple sclerosis Fingolimod and Siponimod, administered (orally or intravenously) to the recipient, three or four times within one week of transplantation (contingent on the simultaneous inhibition of the T-cell dependent acute rejection), abrogate chronic rejection of mouse and rat cardiac allografts (Figure 5).

It must be noted here that Fingolimod, besides inhibiting the RhoA pathway, also inhibits the mTORC2. The mTORC2, in contrast to the mTORC1, which mainly controls cell proliferation, growth, and metabolism, co-regulates, together with the RhoA, actin polymerization [4,73,74,75]. Thus, the effects of Fingolimod on th actin cytoskeleton may be a sum of RhoA and mTORC2 inhibition.

Looking further into the cellular and molecular mechanisms of this anti-chronic rejection effect, we performed a series of in vitro experiments on the cultured mouse/rat macrophages. In addition, using the Lyz 2-Cre/LoxP system, where the Cre recombinase expression is controlled by the monocyte/macrophage-specific Lyz2 promoter, we deleted RhoA from the monocytes/macrophages of the mouse transplant recipient [4,76].

**Figure 6:**
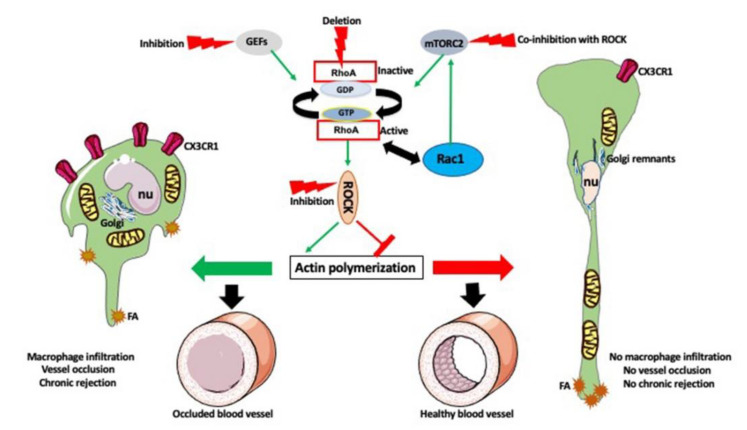
Effect of the RhoA pathway inhibition on macrophages and chronic rejection. RhoA is regulated by GEFs. It is also affected by the mTORC2 pathway and reciprocally interacts with Rac1, which in turn affects the mTORC2 pathway. All these pathways regulate actin polymerization and actin-dependent functions of macrophages. After transplantation, the macrophages infiltrate the graft and cause vessel occlusion, fibrosis, and chronic rejection (**left panel**). Inhibition of RhoA pathway (or co-inhibition of RhoA and mTORC2), or macrophage specific deletion of RhoA, disrupts actin polymerization and actin-dependent functions, causes extreme elongation (hummingbird phenotype), dispersion of the Golgi, and relocation of the nucleus (nu) and mitochondria toward the tail. It also inhibits the expression of CX3CR1 receptors and aggregates the focal adhesions (FA) at the tip of the tail. All these changes prevent macrophage movement into the graft and prevent vessel occlusion and chronic rejection (**right panel**).

The ischemia-reperfusion injury of the harvested organ, and the immune response after the transplantation, induce endothelial cells of the graft’s blood vessel to secrete various chemokines, including fractalkine (CX3CL1), which recruit monocytes and macrophages into the vicinity of blood vessels. Upon arrival, macrophages induce tissue fibrosis and over-proliferation of vessel walls, destroying tissue integrity, and clogging the blood vessel lumen. This, in time, causes chronic rejection and failure of the transplant. We showed that RhoA inhibition or deletion from the recipient’s macrophages prevents macrophage entry into the graft and abrogates or lessens chronic rejection. Further studies showed that the Rho A-deleted macrophages had lower expression of fractalkine receptors (C3CRX3), which prevented macrophages from proper sensing of fractalkine signal released from the graft. The under-expression of the receptors was caused by the faulty distribution of the endocytic vesicles, and disruption of the actin-dependent recycling of the receptors [4]. Studies of the effects of RhoA inhibition or deletion on the macrophage actin cytoskeleton showed that these macrophages were extremely elongated (the so-called hummingbird phenotype). While the length of the control macrophages was around 50 to 60 µm, the hummingbird macrophages were 250 to 750 µm long, and often had one or more breaks in the extremely elongated tail. We showed that the extreme elongation was caused by the disruption of actin-dependent focal adhesion distribution and dynamics, leading to the aggregation of the adhesions at the tip of the tail, and the inability of the moving macrophage to detach the tail. While the front of the macrophage tried to move forward, the tail remained fixed to the substrate, causing extreme elongation and, often, breakage of the tail [4,53]. Interestingly, similar disruption of the actin cytoskeleton and hummingbird phenotype is also caused by the magnetic field gradient forces applied to the macrophages [77,78]. This is not surprising in the light of the recent discovery that RhoA/actin pathway is mechanosensitive [79], and thus, responds to the tension applied to the cell membrane, and that the cytoskeleton transmits and adapts to the mechanical cues [80]. In vitro studies on mouse and rat macrophages treated with RhoA/ROCK inhibitor Y2763 showed a disruption of the actin cytoskeleton that leads not only to the elongation but also, often, to a random displacement of the cell nucleus from the macrophage front to the body or the tail (see Figures 1 and 2 in [81]). We also showed that the changes in actin organization induced by the RhoA interference (inhibition of the upstream regulators of RhoA, GEFs, using Rhosin or Y16 inhibitors, the inhibition of ROCK1 using the Y2762 inhibitor, or the genetic deletion of RhoA) caused the dispersion of the Golgi cysternae and the relocation of the remnants toward the macrophage tail region. Sometimes also the mitochondria were relocated into the macrophage tail (Figure 6); In [4,27,52,53,81,82,83].

All these data indicate that the RhoA interference also affects actin-dependent organization and anchoring of the organelles within the macrophage cytoplasm. The motility, phagocytosis and matrix degradation assays performed on macrophages grown in the presence of Y2763 inhibitor showed that RhoA/ROCK inhibition also causes a decrease in macrophage motility and phagocytosis, and increased matrix degradation [53]. It is possible that the increase in matrix degradation is caused by the fact that the slow moving macrophages stay longer in place and digest more extracellular matrix, or in the change of the podosome arrangement observed both in Y2763 and Fingolimod treatment [52,53]. All these results indicate that the direct or indirect interference with the RhoA pathway has a profound effect on macrophage morphology, and all actin-dependent functions and organelles.

Surprisingly, our studies also showed that the response of macrophages to the RhoA inhibition vary depending on the macrophage subtype. The shape of macrophages is different depending on the subtype: the naïve M0 macrophages are slightly elongated, the inflammatory M1 macrophages are roundish, and the antinflammatory M2 macrophages are elongated. We compared the effect of Rho pathway interference (RhoA deletion or inhibition) on the shape, polarity and expression of subtype-specific molecular markers in the bone-marrow-derived and in vitro polarized macrophages. We showed that the RhoA pathway interference induced hummingbird phenotype in M0 and M2, but not in M1 macrophages. It also inhibited the expression of M2-specific, but not M1-specific molecular markers [81].

All these studies show that the effect of RhoA inhibition is multifactorial and multifaceted, and also suggest that the efficacy of the therapies involving RhoA pathway inhibitors may depend on the subtype of macrophages involved in the different steps of development of a particular disorder. Nevertheless, it seems reasonable to suggest that the macrophage targeted RhoA inhibition therapies should be effective in the treatment of a variety of illnesses and disorders dependent on or affecting the macrophage immune response.

It should be emphasized here that, very often, in many cellular processes the actin filaments function in unison with other cytoskeletal elements, the microtubules [84]. While the globular actin requires ATP to polymerize into filaments, the microtubules self-assemble, in the presence of GTP, from the tubulin dimers. The microtubules are thicker and more rigid than the actin filaments and play a crucial role in cell movement and cell adhesion. The microtubules can be crosslinked to the actin filaments by the multiprotein complexes [84]. Such a crosslinking orients microtubule growth along the actin filaments facilitating directional cell migration and regulating cell shape and rigidity, which are the crucial aspects in the monocyte/macrophage migration from the blood vessel wall into the transplanted organs. There are also examples of microtubule-mediated nucleation of actin filaments in the macrophages. In addition to these direct physical interactions, the actin filaments and microtubules share the regulatory molecules and pathways, which add another dimension to their crosstalk. Both microtubules and actin filaments are regulated by RhoA and other small GTPases, and the microtubule can also regulate RhoA activity and thus actin, by interacting with GEFs and GAPs [84]. This highly complex crosstalk between the microtubules and actin filaments has to be taken into account when, for example, the RhoA pathway inhibitors are applied for the inhibition of chronic rejection of transplanted organs.

## 10. Timing Is the Essence

There are important time-related aspects in the inhibition of chronic rejection of transplanted organs.

The main factor determining the long-term outcome of the transplant is the time of the entry of macrophages to the transplanted organ. It is known that the macrophages enter the transplant within approximately one week of post-transplantation. We showed in the rodent cardiac transplantation models that the administration of one to four doses of RhoA/ROCK inhibitors within seven days post-transplantation inhibits chronic rejection. This indicates that the early blockage of the monocyte recruitment from the circulation is the crux. However, because of the tremendous disparity in the longevity between the rodents and humans (months/years in rodents versus decades in humans), it is very hard to extrapolate this timeline to human transplantation. Although this requires further clinical studies, it is probable that in humans, this early intervention, instead of several days, should last several months post-transplantation. Another time-related issue is that chronic rejection develops and progresses very slowly, within weeks in rodents and months/decades in humans. So, even assuming that the early macrophage entry into the graft is not fully preventable, and a certain number of the monocytes/macrophages will still infiltrate the transplant, there should be a time window for the supplementary anti-macrophage intervention that would inhibit the residual activities of macrophages such as the activation of the pro-fibrotic and vessel wall over-proliferation pathways. It is also possible that the fibrotic and vessel occlusion pathways are not fully coordinated in time and they should be intervened at different time points. Thus, this may require a two-phased delayed intervention. So far, both in rodents and in humans, the exact timing of such a delayed intervention(s) as an additive to the early intervention remains uncharted territory.

There are also time-related events in the monocyte/macrophages at the molecular level. The monocyte/macrophage recruitment and movement depend on the time-coordinated actin polymerization events. Continuous observation and time-lapse imaging of the actin filament formation in the macrophages, such as the nucleation of new actin filaments and the kinetics of filament growth in the presence and absence of RhoA/ROCK inhibitor(s) would pinpoint the exact timing for the inhibition of actin polymerization in the macrophages. Recent advances in the fluorescent dyes, fluorescence microscopy, and the image analysis software, allowing the labeling of actin subunits, the imaging of the single actin filaments, and the documenting of the timeline of elongation and the treadmilling of actin filaments, while eliminating the interference from the unlabeled actin monomers [85], will allow studying the actin polymerization and macrophage movement in real-time.

Another important but rarely thought about this issue is the circadian rhythm of the immunological response and the related diurnal fluctuations of the activity of the immune cells. It is known that the circadian clock coordinates in the time-of-day dependent manner the homeostatic and the innate and adaptive functions of the immune system [86,87,88,89]. Recent studies indicate that immune cell processes such as cell activation, differentiation, chemotaxis, movement, and cytokine release are regulated in a time of day–dependent manner. The circadian oscillations also regulate the immune cell count. Thus, it is not surprising that there is also circadian rhythmicity in the metabolism, expression of the inflammatory molecules, pathogen detection and phagocytosis, and migration and recruitment of the monocytes and macrophages [89]. The circadian oscillations are also tissue-specific, for example, a specific regulation of the expression of adhesion molecules and chemokines by the endothelial cells [90]. This indicates that also the recruitment of the monocytes/macrophages by the vessel wall endothelium during the development of chronic rejection is regulated by circadian rhythmicity. This, in turn, might influence the effectiveness of the anti-chronic rejection therapies and calls for the application of circadian rhythm discoveries in the immune response field into modified therapeutic interventions for the management of chronic rejection, which account for the diurnal oscillations of the patients’ responses to medication. The potential effects of the circadian rhythm on the transplantation outcome are the time of the day when the transplantation is performed, the timing (within 24 h) of the optimal function of the transplanted organ, and the circadian rhythm disorder, such as sleep disorder, or the night-shift working hours of the organ donor. Although this is a recently emerging theme and the studies on the effects of circadian rhythm on transplantation outcome are extremely sparse [91], the future pre-transplantation assessments of the blood and tissue type, organ size, and health might also need to include the circadian clock-related parameters.

## 11. Conclusions and Future Approaches

Although the role of macrophages and RhoA pathway and the actin cytoskeleton in the development of the chronic rejection of transplanted organs is well documented in animal studies, their role in human transplantation is still underappreciated. Finding the clinically applicable anti-chronic rejection therapy will require extensive clinical trials and the development of novel clinically applicable RhoA inhibitors or the reformulation of the existing drugs. Similarly underappreciated is the effect of the biological clock and circadian rhythm on the transplant donor and recipient, the fitness of the transplanted organ, and the time of the day when the transplantation procedure is performed. At present, the assessment of these parameters in humans is impossible, it would require the identification of the clinically applicable biological clock/circadian rhythm-related markers.

## Data Availability

Not applicable.
